# Association of *MTHFR* and platelet-related blood cell ratio parameters with ischemic stroke in Gansu population

**DOI:** 10.1186/s12872-026-06229-2

**Published:** 2026-07-07

**Authors:** Youli Zhao, Xiaojuan Li, Yijie Wang, Mengdan Song

**Affiliations:** 1 Department of Clinical Medical Laboratory, The Second Hospital & Clinical Medical School, Lanzhou University, Lanzhou, Gansu 730030 China; 2 Department of Clinical Transfusion Medicine, The Second Hospital & Clinical Medical School, Lanzhou University, Lanzhou, Gansu 730030 China; 3 Department of Transfusion Medicine, Xigu Hospital of Lanzhou University Second Hospital, Lanzhou, Gansu 730060 China

**Keywords:** Ischemic stroke, *MTHFR*, Blood cell-based ratio, Gansu region of China

## Abstract

**Objective:**

To investigate the association of MTHFR polymorphisms with the susceptibility to ischemic stroke (IS) in Gansu population in China, and the predictive value of platelet-related ratios for the risk of IS in different genotypes.

**Methods:**

We enrolled 121 IS patients admitted between June and December 2022 and 121 recruited from the same source population healthy controls. Clinical data were collected, and peripheral blood was analyzed for routine parameters. Genotyping for MTHFR C677T and A1298C was performed using PCR fluorescence probe. Statistical analysis was conducted with SPSS Statistics 27.0.

**Results:**

In the Gansu population, the MTHFR C677T TT and A1298C CC genotypes were significantly associated with increased IS risk (*P* = 0.005, *P* = 0.01). Specific combined genotypes at these loci (CT/CC, TT/AC, TT/CC) elevated IS risk by 1.4-, 1.7- and 1.4-fold, respectively. In the IS group, the MPV/PLT, MPV/LY and RDW/PLT ratios were significantly elevated, while PLT/NE was lower (all *P* < 0.05). Additionally, patients with the MTHFR C677T TT genotype had higher NE levels than those with the CC genotype (*P* = 0.028), and those with the MTHFR A1298C CC genotype exhibited a higher MPV/LY ratio than AA carriers (*P* = 0.038). ROC analysis indicated that combining NE and MPV/LY for IS risk assessment yielded an AUC of 0.784 (95% CI: 0.726–0.842).

**Conclusion:**

MTHFR C677T and A1298C polymorphisms are associated with IS risk in the Gansu population. The combined assessment of NE, MPV/LY, and MTHFR C677T genotyping may offer adjunctive information for IS risk assessment, pending independent validation.

**Supplementary Information:**

The online version contains supplementary material available at https://doi.org/10.1186/s12872-026-06229-2.

## Introduction

Stroke is the second leading cause of death and the third leading cause of disability worldwide, with ischemic stroke (IS) accounting for approximately 65.3% of all stroke cases [1]. Low- and middle-income countries bear the heaviest burden, contributing to 77% of stroke cases, 87% of stroke-related deaths, and 89% of disability-adjusted life years lost [2–4]. Early identification of high-risk individuals and timely intervention are key strategies to reduce this burden [5].

The pathogenesis of IS is complex and multifactorial. In addition to traditional risk factors such as hypertension, diabetes, and dyslipidemia, genetic factors are increasingly recognized to play a significant role in stroke risk and prognosis [6, 7]. However, genetic loci identified by genome-wide association studies to date do not fully explain the heritable risk of IS [8–12]. Meta-analyses suggest that different stroke subtypes may have distinct genetic backgrounds and pathological mechanisms [6, 13], highlighting the need for population-specific genetic studies to enable personalized prevention and treatment [14].

MTHFR (methylenetetrahydrofolate reductase) is a key enzyme in folate and homocysteine metabolism. The MTHFR C677T (rs1801133) and A1298C (rs1801131) polymorphisms are known to reduce enzyme activity, which may lead to elevated homocysteine levels-an independent risk factor for IS [15, 16]. Although the association between MTHFR polymorphisms and IS risk has been investigated in various populations, findings across different ethnic groups remain inconsistent. In the Chinese population, the allele distributions of MTHFR C677T and A1298C exhibit unique ethnic and geographical characteristics [17–19]. However, studies focusing on the Gansu population—a multi-ethnic region in northwestern China inhabited by Han, Hui, Tibetan, Dongxiang, Yugur and Bonan populations—are lacking.

In recent years, composite blood cell ratio parameters, including mean platelet volume to platelet count ratio (MPV/PLT), mean platelet volume to lymphocyte count ratio (MPV/LY), platelet count to neutrophil count ratio (PLT/NE), and red cell distribution width to platelet count ratio (RDW/PLT), have emerged as novel indicators of inflammation and thrombosis. Compared with traditional single parameters, these composite ratios more sensitively capture dynamic multi-system imbalances and have shown advantages in risk stratification and early warning for cardiovascular and cerebrovascular diseases [20–24].

This study aimed to investigate the association between MTHFR C677T and A1298C polymorphisms and IS risk, and to explore the predictive value of combining these genetic markers with platelet-related blood cell ratios. The findings are intended to provide a scientific basis for identifying high-risk individuals and enabling personalized prevention of IS in this understudied population.

## Materials and methods

### Study design and population

This was a retrospective case-control study. A total of 121 patients with acute ischemic stroke admitted to The Second Hospital & Clinical Medical School of Lanzhou University from June 2022 to December 2022 were enrolled as the case group. The diagnosis of ischemic stroke was based on the Chinese Guidelines for the Diagnosis and Treatment of Acute Ischemic Stroke 2018 issued by the Cerebrovascular Disease Group of the Neurology Branch of the Chinese Medical Association [25]. The case group included 79 males and 42 females, aged 35–88 years (mean 66 ± 12 years).

The control group consisted of 121 healthy volunteers recruited from the same hospital’s physical examination center during the same study period (June–December 2022). Controls were not strictly matched to cases by age or gender, which we acknowledge as a limitation. The control group included 65 males and 56 females, aged 37–81 years (mean 56 ± 8 years). All controls received standardized physical examinations and medical history interviews; individuals with a history of cerebrovascular disease, coronary heart disease, or other major cardiovascular diseases were strictly excluded.

Exclusion criteria for patients were: ① cerebral hemorrhage or subarachnoid hemorrhage confirmed by CT or MRI; ② expected lifespan less than 3 months (e.g., advanced malignancy) or severe cardiopulmonary, hepatic, or renal insufficiency; ③ other diseases seriously affecting neurological function assessment (e.g., severe dementia, advanced Parkinson’s disease); ④ use of medications that may affect MPV, PLT, RDW, LY, or NE levels (e.g., antiplatelet drugs, statins, non-steroidal anti-inflammatory drugs), as determined by standardized medical record review.

This study was approved by the Hospital Ethics Committee (Approval No. 2024 A-239) and was conducted in accordance with the Declaration of Helsinki. Informed consent was waived due to the retrospective nature of the study.

### Sample size calculation

Sample size was estimated based on a prior meta-analysis of the MTHFR C677T polymorphism and ischemic stroke risk in the Chinese population, which reported a pooled OR of approximately 1.6 for the TT genotype with a TT genotype prevalence of approximately 25% in controls [15]. With α = 0.05 and power = 0.80, the required minimum sample size was calculated to be 110 per group. We enrolled 121 cases and 121 controls, which provided adequate statistical power.

### Genotyping

Genomic DNA was extracted from EDTA-anticoagulated peripheral blood using the Natch96 Nucleic Acid Extraction Instrument (Sansure Biotech Inc., Changsha, China) with the matching extraction kit.

Genotyping was performed using the Human MTHFR Gene Polymorphism Detection Kit (Sansure Biotech Inc., Changsha, China) on a Bio-Rad CFX96 Real-Time PCR System (Bio-Rad Laboratories, Hercules, CA, USA). The kit contains pre-designed primers and fluorescent probes for MTHFR C677T (rs1801133) and MTHFR A1298C (rs1801131), with β-actin as an internal control. The primer and probe sequences are provided in Supplementary Table S5.

Each 20 µL PCR reaction contained 10 µL master mix, 1 µL probe/primer mix, 2 µL genomic DNA (20–50 ng), and 7 µL RNase-free water. Thermal cycling conditions were: 50 °C for 2 min, 95 °C for 10 min, followed by 40 cycles of 95 °C for 15 s and 60 °C for 1 min. For quality control, 10% of the samples were randomly selected and re-genotyped for reproducibility, yielding a concordance rate of 100%. Negative controls (no template) were included in each PCR run.

### Routine blood tests and ratio calculation

EDTA-anticoagulated venous blood was collected from each participant. Routine blood parameters, including platelet count (PLT), mean platelet volume (MPV), red blood cell distribution width (RDW), lymphocyte count (LY), and neutrophil count (NE), were measured using a Mindray BC-6801 Automatic Hematology Analyzer (Mindray Bio-Medical Electronics Co., Ltd., Shenzhen, China) with matching proprietary reagents. All measurements were completed within 2 h of blood collection. The analyzer was calibrated daily using the manufacturer’s standard controls, and internal quality control samples were run with each batch.

The following ratios were subsequently calculated:

$$\begin{aligned}\:\mathrm{MPV}/\mathrm{PLT}\;&=\;\mathrm{mean}\:\mathrm{platelet}\:\mathrm{volume}\:\\&/\:\mathrm{platelet}\:\mathrm{count}\end{aligned}$$

$$\begin{aligned}\:\mathrm{MPV}/\mathrm{LY}\;&=\;\mathrm{mean}\:\mathrm{platelet}\:\mathrm{volume}\:\\&/\:\mathrm{lymphocyte}\:\mathrm{count}\end{aligned}$$

$$\mathrm{PLT}/\mathrm{NE}=\mathrm{platelet}\:\mathrm{count}\:/\:\mathrm{neutrophil}\:\mathrm{count}$$

$$\mathrm{RDW}/\mathrm{PLT}=\mathrm{red}\:\mathrm{blood}\:\mathrm{cell}\:\mathrm{distribution}\:\mathrm{width}\:/\:\mathrm{platelet}\:\mathrm{count}$$

### Statistical analysis

Data analysis was performed using IBM SPSS Statistics 27.0. Normally distributed continuous data were expressed as mean ± standard deviation and compared between groups using the independent samples t-test. Categorical data were expressed as frequency (n) and percentage (%) and compared using the chi-square test or Fisher’s exact test (when expected cell counts were < 5). Hardy-Weinberg equilibrium was tested for each genetic locus in both groups using the chi-square test.

Multivariate binary logistic regression was performed to adjust for potential confounders (age and gender) and to calculate adjusted odds ratios (ORs) with 95% confidence intervals (CIs) for the association between each genotype and IS risk.

To account for multiple comparisons, Bonferroni correction was applied. For the two main genetic comparisons (MTHFR C677T and A1298C), the corrected significance threshold was set at α = 0.025 (0.05/2). For the nine combined genotype comparisons, the threshold was α = 0.0056 (0.05/9). Corrected *P*-values are reported in the corresponding tables.

For the combined ROC analysis, binary logistic regression was performed with ischemic stroke status as the dependent variable and NE, MPV/LY, and MTHFR C677T genotype (coded as 0 for CC, 1 for CT, and 2 for TT) as independent variables. The predicted probability of being a case was saved and used as the test variable to construct the ROC curve. The area under the curve (AUC) was then calculated to evaluate the combined predictive value of these three variables.

A two-tailed *P*-value < 0.05 was considered statistically significant unless otherwise specified for multiple comparisons.

## Results

### Baseline characteristics

A total of 242 subjects were enrolled, including 121 in the ischemic stroke group (79 males, 42 females; mean age 66 ± 12 years) and 121 healthy controls (65 males, 56 females; mean age 56 ± 8 years). As shown in Table 1, the IS group had a significantly higher mean age than the control group (mean difference = 10.0 years, 95% CI: 9.68–10.34, *P* < 0.001). Gender distribution did not differ significantly between the two groups (*P* = 0.067). Compared with controls, the IS group had significantly higher levels of MPV (*P* < 0.001), RDW (*P* = 0.001), and NE (*P* < 0.001), and a significantly lower LY level (*P* < 0.001). No significant difference in PLT level was observed between the two groups (*P* = 0.156).

**Table 1:** Demographic and clinical characteristics of the study population

Variables	Ischemic Stroke(*n* = 121)	Control(*n* = 121)	*P*-value
Age(years) mean ± SD	66 ± 12	56 ± 8	0.000
Gender, *n* (%)			
Male	79(65.29%)	65(53.72%)	0.067
Female	42(34.71%)	56(46.28%)	
NE(×10^9^/L)	5.59 ± 3.58	3.43 ± 1.15	<0.001***
LY (×10^9^/L)	1.41 ± 0.67	1.73 ± 0.60	<0.001***
PLT(×10^9^/L)	192.27 ± 70.75	203.59 ± 51.35	0.156
MPV (fL)	11.37 ± 1.13	10.49 ± 1.39	<0.001***
RDW	46.04 ± 6.39	43.92 ± 2.73	0.001***

Data are presented as mean ± standard deviation or *n* (%)

*** *P* < 0.001

### Association of MTHFR polymorphisms with ischemic stroke risk

#### Hardy–weinberg equilibrium

Genotype distributions of MTHFR C677T and MTHFR A1298C in both the IS and control groups conformed to Hardy–Weinberg equilibrium (all *P* > 0.05; see Supplementary Table 7 for exact P‑values).

#### Genotype and allele distributions

**Fig. 1:**
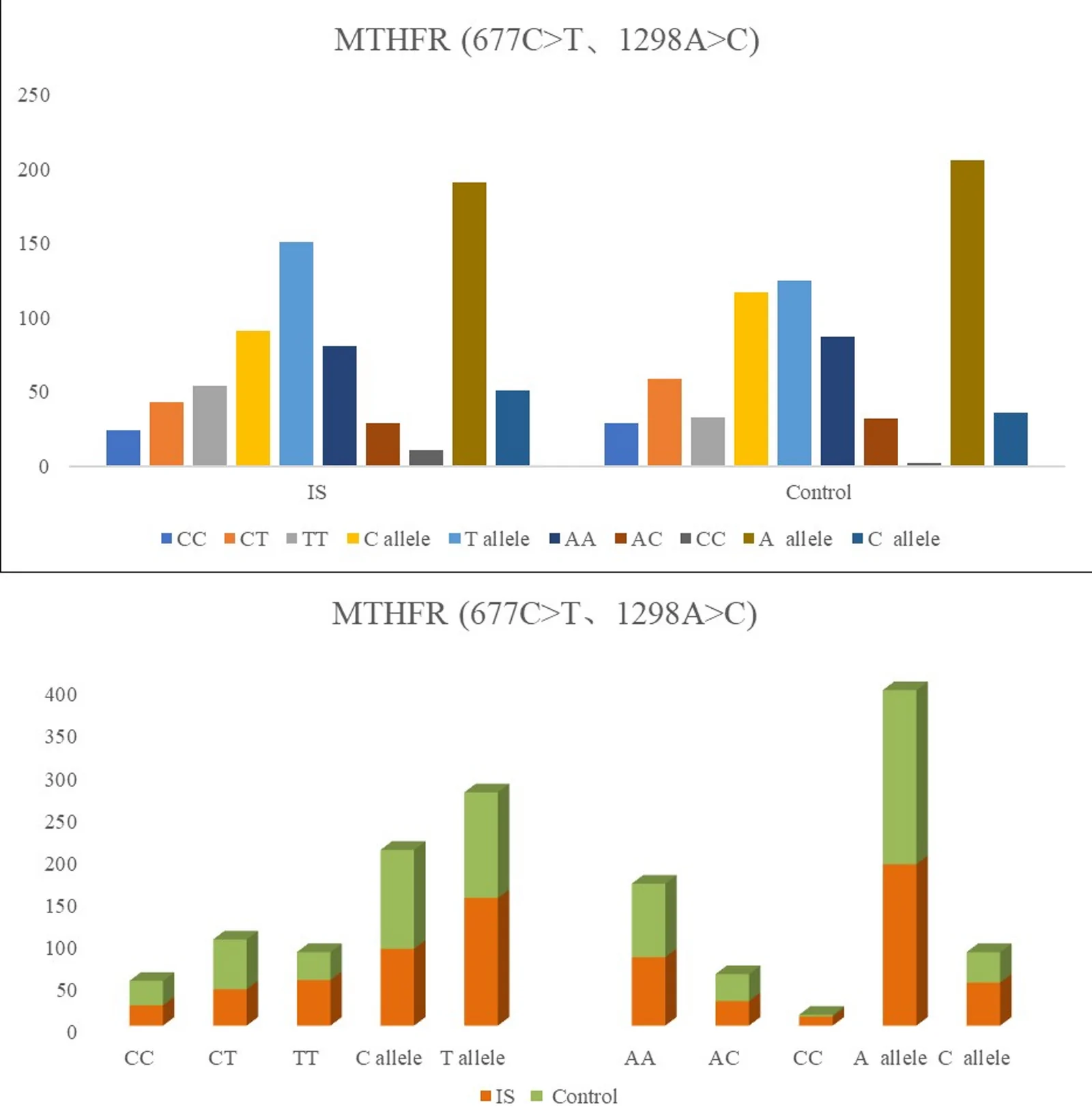
MTHFR C677T and A1298C genotype distribution

As shown in Table 2, the distributions of MTHFR C677T genotypes differed significantly between the IS and control groups (*P* = 0.018). The frequency of the TT genotype was 44.63% in the IS group versus 27.27% in controls, and the T allele frequency was 62.40% vs. 51.65% (*P* = 0.017). For MTHFR A1298C, the genotype distribution also differed significantly (*P* = 0.037), with the CC genotype present in 9.09% of IS patients but only 1.65% of controls. The C allele frequency was 21.07% in the IS group versus 14.88% in controls, although this difference did not reach statistical significance (*P* = 0.076). Figure 1 illustrates the genotype distribution visually.

**Table 2:** MTHFR C677T and A1298C genotype distribution

Genotype		Case (%)	Control (%)	*P*-value
*MTHFR (677 C > T)*				
	CC	24(19.83)	29(23.97)	0.018*
	CT	43(35.54)	59(48.76)	
	TT	54(44.63)	33(27.27)	
	C allele	91(37.60)	117(48.35)	0.017*
	T allele	151(62.40)	125(51.65)	
*MTHFR (1298 A > C)*				
	AA	81(66.94)	87(71.90)	0.037*
	AC	29(23.97)	32(26.45)	
	CC	11(9.09)	2(1.65)	
	A allele	191(78.93)	206(85.12)	0.076
	C allele	51(21.07)	36(14.88)	

Data are presented as *n* (%)

* *P* < 0.05

#### Gender‑stratified and interaction analyses

Formal genotype‑by‑gender interaction tests (Table 3) revealed no significant effect modification by gender for MTHFR C677T (P for interaction = 0.184) or MTHFR A1298C (P for interaction = 0.421), indicating that the associations between these polymorphisms and IS risk are not significantly modified by gender.

**Table 3:** Genotype-by-gender interaction analysis for MTHFR polymorphisms

Locus	Genotype comparison	Group	Male (*n* = 144)	Female (*n* = 98)	*P* for interaction
*MTHFR C677T*	TT vs. CC + CT	Ischemic stroke	37/79 (46.83%)	17/42 (40.48%)	0.184
		Control	16/65 (24.62%)	17/56 (30.36%)	
*MTHFR A1298C*	CC vs. AA + AC	Ischemic stroke	9/79 (11.39%)	2/42 (4.76%)	0.421
		Control	1/65 (1.54%)	1/56 (1.79%)	

Data are presented as *n* (%)

#### Multivariate regression and risk association

Multivariate logistic regression adjusting for age and gender was performed to assess the independent associations. As shown in Table 4, after adjustment, the MTHFR C677T TT genotype remained significantly associated with an increased risk of IS (adjusted OR = 2.149, 95% CI: 1.26–3.68, *P* = 0.005), as did the MTHFR A1298C CC genotype (adjusted OR = 5.95, 95% CI: 1.29–27.45, *P* = 0.01). After Bonferroni correction for two comparisons (α = 0.025), both associations remained significant (corrected *P* = 0.005 and *P* = 0.02, respectively).

**Table 4:** Multivariate analysis of MTHFR polymorphisms and IS risk

Locus	Genotype comparison	Adjusted OR*	95% CI	*P*-value	*P*-value (Bonferroni-corrected)
*MTHFR C677T*	677 TT vs. CC + CT	2.149	1.26–3.68	0.005	0.005**
*MTHFR A1298C*	1298 CC vs. AA + AC	5.95	1.29–27.45	0.01	0.02*

Bonferroni correction for 2 comparisons (α = 0.025). Adjusted for age, gender

* *P* < 0.05, ** *P* < 0.01

#### Combined genotype analysis of MTHFR C677T and A1298C

The combined effects of the two polymorphisms are presented in Table 5. Compared with the CC/AA reference group, the CT/CC, TT/AC and TT/CC combined genotypes were significantly more frequent in the IS group (*P* = 0.031, *P* = 0.006 and *P* = 0.031, respectively), corresponding to ORs of 1.400 (95% CI: 1.005–1.950), 1.700 (95% CI: 1.142–2.530) and 1.400 (95% CI: 1.005–1.950), respectively. After Bonferroni correction for nine comparisons (α = 0.0056), only the TT/AC combination remained statistically significant (corrected *P* = 0.009); the CT/CC and TT/CC combinations showed corrected *P* = 0.098, which we interpret as suggestive rather than definitive.

**Table 5:** Different genotype combinations of MTHFR C677T and MTHFR A1298C polymorphisms

Genotypes	Control (*n*)	Ischemic Stroke (*n*)	OR (95% CI)	*P*	*P*-value (Bonferroni-corrected)
CC/AA	14	10	1.000(reference)	-	
CC/AC	13	11	1.185(0.378–3.710)	0.771	1.00
CC/CC	2	3	2.100(0.294–14.978)	0.453	0.632
CT/AA	40	28	0.980(0.381–2.520)	0.967	1.00
CT/AC	19	11	0.811(0.270–2.435)	0.708	0.783
CT/CC	0	4	1.400(1.005–1.950)	0.031*	0.098
TT/AA	33	43	1.824(0.720–4.622)	0.202	0.244
TT/AC	0	7	1.700(1.142–2.530)	0.006**	0.009**
TT/CC	0	4	1.400(1.005–1.950)	0.031*	0.098

Bonferroni correction for 9 comparisons (α = 0.0056). Data are presented as *n* (%)

* *P* < 0.05, ** *P* < 0.01

### Platelet‑related blood cell ratios and ischemic stroke risk

As shown in Table 6, the IS group had significantly higher MPV/PLT (0.07 ± 0.04 vs. 0.06 ± 0.02, *P* = 0.001), MPV/LY (10.22 ± 6.05 vs. 6.68 ± 2.27, *P* < 0.001) and RDW/PLT ratios (0.28 ± 0.14 vs. 0.23 ± 0.08, *P* = 0.002) compared with controls. In contrast, the PLT/NE ratio was significantly lower in the IS group (45.48 ± 27.73 vs. 65.19 ± 26.39, *P* < 0.001).

**Table 6:** Comparison of clinical characteristics between the study and control groups

Variables	Ischemic Stroke	Control	*P*-value
MPV/PLT	0.07 ± 0.04	0.06 ± 0.02	0.001***
MPV/LY	10.22 ± 6.05	6.68 ± 2.27	0.000***
PLT/NE	45.48 ± 27.73	65.19 ± 26.39	0.000***
RDW/PLT	0.28 ± 0.14	0.23 ± 0.08	0.002***

Data are presented as mean ± standard deviation

*** *P* < 0.001

### Effect of MTHFR genotypes on blood cell parameters and ratios

Table 7 summarizes the relationships between MTHFR genotypes and blood cell parameters in IS patients. Within the IS group, patients with the MTHFR C677T TT genotype had significantly higher NE levels than those with the CC or CT genotype (*P* = 0.028). For MTHFR A1298C, patients carrying the CC genotype exhibited a significantly higher MPV/LY ratio than those with the AA or AC genotype (*P* = 0.038). No other significant associations were observed for the other parameters or ratios (all *P* > 0.05).

**Table 7:** Relationship between MTHFR genotype distribution and relative risk of ischemic stroke

Variables	Biomarker	MTHFR C677T			MTHFR A1298C		
		CC	CT	TT	AA	AC	CC
Control	NE	3.39 ± 1.18	3.40 ± 1.21	3.52 ± 1.06	3.48 ± 1.14	3.28 ± 1.19	3.39 ± 1.63
	LY	1.60 ± 0.42	1.83 ± 0.71	1.68 ± 0.52	1.75 ± 0.62	1.70 ± 0.60	1.68 ± 0.21
	RDW	44.41 ± 2.63	43.97 ± 2.86	43.39 ± 2.55	43.93 ± 2.65	43.88 ± 3.03	44.00 ± 1.41
	PLT	207.07 ± 62.76	205.15 ± 50.82	197.73 ± 41.35	200.40 ± 46.30	206.00 ± 51.12	303.50 ± 173.24
	MPV	10.53 ± 1.52	10.29 ± 1.23	10.82 ± 1.49	10.53 ± 1.48	10.44 ± 1.15	9.85 ± 1.06
	MPV/PLT	0.06 ± 0.03	0.05 ± 0.02	0.06 ± 0.02	0.06 ± 0.02	0.06 ± 0.02	0.04 ± 0.03
	MPV/LY	6.97 ± 1.88	6.27 ± 2.17	7.15 ± 2.69	6.66 ± 2.33	6.78 ± 2.21	5.89 ± 0.09
	PLT/NE	68.06 ± 33.93	65.39 ± 22.20	62.33 ± 26.35	62.74 ± 22.98	68.75 ± 25.99	115.02 ± 106.29
	RDW/PLT	0.23 ± 0.08	0.23 ± 0.09	0.23 ± 0.06	0.23 ± 0.06	0.24 ± 0.12	0.17 ± 0.10
Ischemic stroke	NE	4.88 ± 2.21	4.93 ± 3.73	6.43 ± 3.83*	5.67 ± 3.82	5.10 ± 2.73	6.31 ± 3.93
	LY	1.56 ± 0.83	1.44 ± 0.63	1.32 ± 0.61	1.39 ± 0.59	1.60 ± 0.85	1.05 ± 0.55
	RDW	45.38 ± 5.90	46.09 ± 6.09	46.30 ± 6.91	46.42 ± 6.99	44.69 ± 4.83	46.82 ± 5.29
	PLT	204.25 ± 55.57	190.40 ± 83.14	188.44 ± 66.58	194.07 ± 73.22	195.97 ± 60.53	169.27 ± 79.21
	MPV	11.17 ± 1.09	11.39 ± 1.25	11.44 ± 1.06	11.45 ± 1.14	11.16 ± 1.09	11.33 ± 1.21
	MPV/PLT	0.06 ± 0.03	0.08 ± 0.05	0.07 ± 0.03	0.07 ± 0.03	0.07 ± 0.05	0.08 ± 0.04
	MPV/LY	9.17 ± 5.75	9.67 ± 4.96	11.12 ± 6.89	10.17 ± 5.88	8.80 ± 5.10	14.33 ± 8.09*
	PLT/NE	50.25 ± 24.63	49.03 ± 27.02	40.54 ± 29.22	45.11 ± 27.51	49.52 ± 29.26	37.60 ± 25.66
	RDW/PLT	0.25 ± 0.11	0.30 ± 0.18	0.27 ± 0.09	0.27 ± 0.12	0.27 ± 0.16	0.34 ± 0.17

Data are presented as mean ± standard deviation

* *P* < 0.05, *Significant differences (*P* < 0.05) are indicated with asterisks. In the ischemic stroke group, NE levels were significantly higher in patients with MTHFR C677T TT genotype than in those with CC or CT genotype (*P* = 0.028). MPV/LY ratio was significantly elevated in patients carrying MTHFR A1298C CC genotype compared with AA or AC genotype (*P* = 0.038)

### ROC curve analysis for risk prediction

**Fig. 2:**
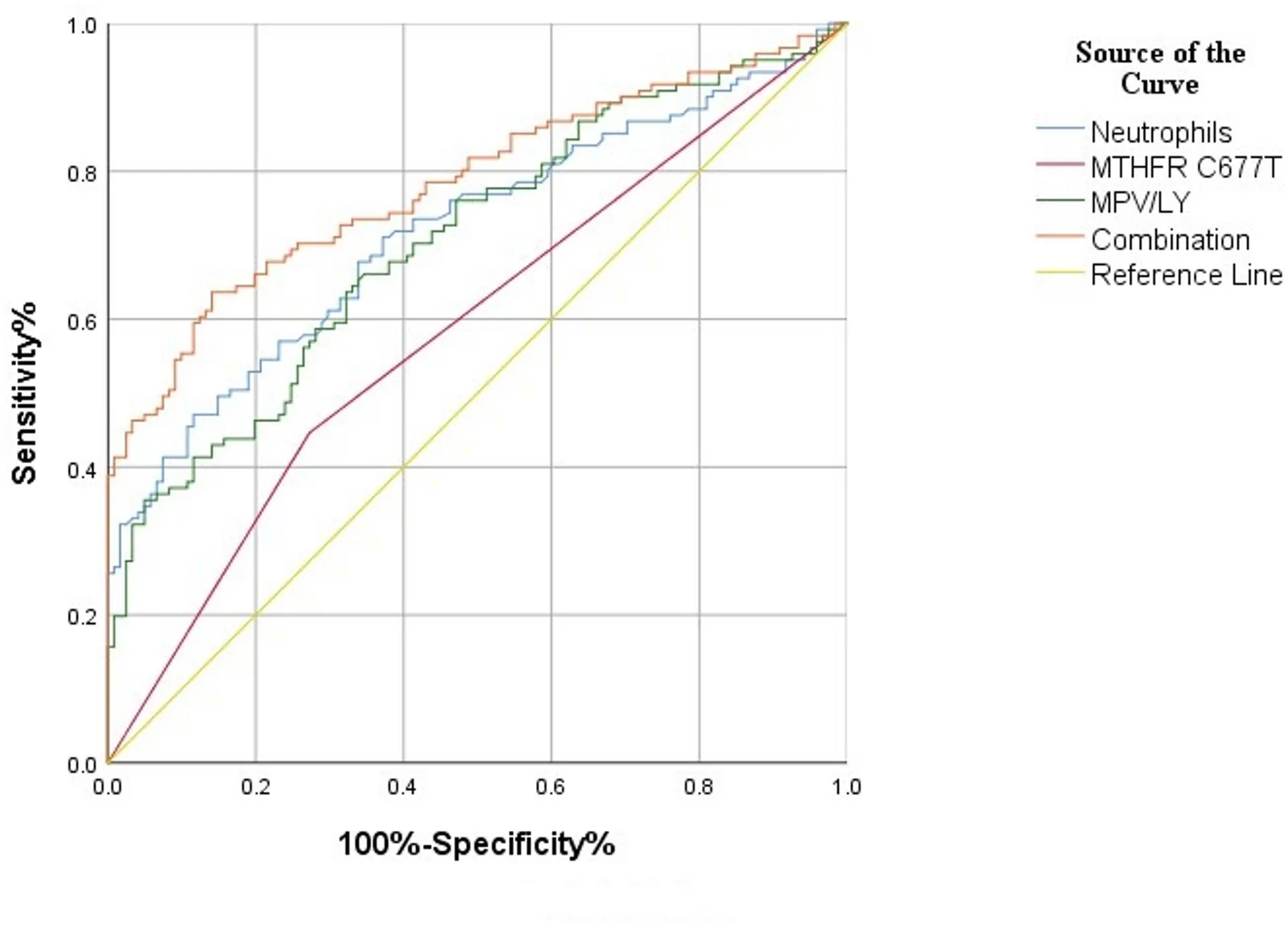
Receiver operating characteristic curve prediction model

Using the predicted probability derived from logistic regression combining NE, MPV/LY and MTHFR C677T genotype, the ROC curve for assessing IS risk yielded an area under the curve (AUC) of 0.784 (95% CI: 0.726–0.842), as shown in Fig. 2. This combined model demonstrated superior predictive performance compared with any single indicator alone.

## Discussion

Accumulating evidence has demonstrated that genetic factors play a crucial role in the pathophysiological processes of ischemic stroke at different progression stages. MTHFR is a gene encoding a key enzyme involved in folate metabolism and homocysteine methylation. Mutations at MTHFR C677T and MTHFR A1298C loci are hypothesized to significantly reduce the activity of methylenetetrahydrofolate reductase (MTHFR), thereby impairing the reduction of 5,10‑methylenetetrahydrofolate to bioavailable 5‑methyltetrahydrofolate in vivo. This may further interfere with the conversion of homocysteine to methionine, potentially leading to elevated blood homocysteine levels—a well‑recognized independent risk factor for ischemic stroke [16, 26]. Such genetic factors may amplify susceptibility to ischemic stroke when combined with other related risk factors, although inconsistent findings have been reported across different ethnicities and populations.

When compared with other Chinese populations, the allele frequencies in our Gansu cohort show notable differences. In our control group, the T allele frequency of MTHFR C677T was 51.65%, which is substantially higher than that reported in a southern Chinese Hakka population (25.11%) [19] and also higher than that reported in a Beijing population‑based cohort (approximately 45%) [27]. This difference likely reflects the known north–south genetic gradient within China, as well as the unique ethnic composition of the Gansu region, which includes Han, Hui, Tibetan, Dongxiang, Yugur and Bonan populations. The higher T allele frequency in our control group suggests that the baseline genetic susceptibility to MTHFR‑related ischemic stroke risk may be elevated in the Gansu population compared with southern Chinese populations. However, the case–control comparison within our cohort still demonstrated a significant association between the TT genotype and IS risk (adjusted OR = 2.149, *P* = 0.005), indicating that even against a background of higher T allele prevalence, the additional risk conferred by the homozygous TT genotype remains detectable and clinically relevant in this population.

A Mendelian randomization study showed that the MTHFR C677T mutation was associated with an increased risk of small vessel stroke in East Asian (OR = 1.20, 95% CI: 1.08–1.34, *P* = 8.58 × 10⁻⁴) and European populations (OR = 1.62, 95% CI: 1.24–2.12, *P* = 3.73 × 10⁻⁴), but not with cardioembolic stroke or large artery stroke [28]. A prospective cohort study in Beijing, China, indicated that elevated serum folate concentrations in individuals with the MTHFR C677T CC genotype were significantly associated with a reduced risk of ischemic stroke (HR = 0.88, 95% CI: 0.80–0.98, *P* = 0.035) [27]. In the Hakka population in southern China, the frequency of the T allele at C677T was significantly higher in the ischemic stroke group (27.17%) than in the control group (25.11%) (*P* = 0.026, OR = 1.113, 95% CI: 1.013–1.223), and the homozygous TT genotype (7.02%) increased the risk of ischemic stroke compared with controls (5.85%) (*P* = 0.049, OR = 1.132, 95% CI: 1.001–1.281) [19]. These findings are consistent with the trend of our results. In our study, the frequency of the T allele was significantly higher in the ischemic stroke group (62.40%) than in the control group (51.65%) (*P* = 0.017), and the homozygous TT genotype (44.63%) was associated with an increased risk of ischemic stroke in univariate analysis (OR = 1.636, 95% CI: 1.151–2.327, *P* = 0.005). After adjusting for age and gender, the association remained significant (adjusted OR = 2.149, 95% CI: 1.26–3.68, *P* = 0.005), and it survived Bonferroni correction (corrected *P* = 0.005).

The MTHFR A1298C polymorphism is located in the 7th exon of the MTHFR gene, where adenine (A) is substituted by cytosine (C), resulting in the replacement of glutamic acid with alanine. Compared with the wild‑type AA genotype, the enzyme activity of the heterozygous AC genotype is approximately 65% of the normal level, and that of the homozygous CC genotype is about 60% of the normal level—its effect on reducing enzyme activity is thought to be much weaker than that of the C677T polymorphism. Numerous large‑scale studies have shown that isolated carriage of the A1298C mutation (especially the heterozygous AC genotype) has a weak association with the risk of ischemic stroke, which may not be sufficient to constitute a significant clinical risk alone [29–32]. When individuals carry both C677T and A1298C mutations, the defect in MTHFR enzyme activity may be amplified, leading to higher homocysteine levels and thus potentially increasing the risk of stroke. Recent meta‑analyses have indicated a significant correlation between MTHFR A1298C and stroke risk in Asian populations, but no correlation in Caucasian and African populations [33]. In the Egyptian population, the proportion of the MTHFR A1298C AC heterozygous genotype was higher in stroke patients [34]. In our study, the CC genotype was associated with a 5.500‑fold increased risk of ischemic stroke in univariate analysis (95% CI: 1.245–24.292, *P* = 0.01). After adjusting for age and gender, the association remained significant (adjusted OR = 5.95, 95% CI: 1.29–27.45, *P* = 0.01), and it also survived Bonferroni correction (corrected *P* = 0.02). When combined with C677T, the proportions of CT/CC, TT/AC and TT/CC genotype combinations were significantly higher in the ischemic stroke group than in the control group. However, after Bonferroni correction for nine comparisons, only the TT/AC combination remained significant (corrected *P* = 0.009); the CT/CC and TT/CC combinations showed corrected *P* = 0.098, which we interpret as suggestive rather than definitive. The relatively modest effect sizes (ORs of 1.400–1.700) and the confidence intervals bordering on 1.0 for some comparisons indicate that these combined genotypes, if truly associated with IS risk, confer only a small increase in susceptibility. These findings are hypothesis‑generating and require independent validation in larger cohorts.

Platelet count (PLT) usually reflects the balance between platelet production and consumption/destruction. Meanwhile, it serves as an important inflammatory mediator, participating in the formation and destabilization of atherosclerotic plaques. Such an unstable intravascular environment is highly prone to promoting the formation and development of thrombosis [35, 36]. When neutrophils respond to pathogens or certain inflammatory stimuli, antimicrobial granular proteins in the cytoplasm mix with depolymerized DNA, which are collectively released into the extracellular space through cell membrane rupture or pore formation. The DNA skeleton of neutrophil extracellular traps (NETs) has strong procoagulant activity, which can directly activate platelets and coagulation factors, acting as the “core scaffold” of thrombi. This plays a crucial role in thrombosis‑related complications such as stroke, deep vein thrombosis, and myocardial infarction [37, 38]. The PLT/NE ratio can reflect the interaction between the inflammatory response and the coagulation system. A decreased PLT/NE ratio implies a significant increase in neutrophils and/or a consumptive decrease in platelets. Studies have shown that a reduced PLT/NE ratio indicates systemic inflammation, severe bacterial infection, and consequent coagulation disorders [39]. In our study, the PLT/NE ratio was significantly lower in the ischemic stroke group than in the control group, suggesting that it may serve as a potential early warning indicator.

In thromboembolic diseases, an elevated MPV usually indicates that more newly generated, larger, younger, and metabolically active platelets are involved in thrombosis. Lymphocytes are important components of the body’s immune defense; changes in lymphocyte subsets can reflect the regulatory state of immune‑inflammatory responses, and lymphopenia is a marker of immune exhaustion or suppression in severe infections, sepsis, or inflammatory storms [40]. The MPV/LY ratio integrates information from two distinct but interrelated pathophysiological pathways (the hemostatic/inflammatory system and the immune system), providing a different perspective for interpreting the disease progression process. Studies have shown that the MPV/LY ratio is associated with the severity of coronary artery lesions, serves as a predictor of poor prognosis in patients with acute myocardial infarction, and may also be associated with the risk of thromboembolism in patients with atrial fibrillation [41–43]. In our study, a high MPV/LY ratio was positively correlated with the risk of ischemic stroke, consistent with these previous findings.

MPV/PLT is a novel indicator that evaluates platelet kinetics and bone marrow function by integrating information on platelet quantity (PLT) and quality (MPV). Studies have suggested that it has important auxiliary diagnostic value for differentiating the etiology of thrombocytopenia and assessing thrombotic diseases and the progression of their conditions [44]. A large number of clinical studies have shown that a higher RDW/PLT ratio at admission is closely associated with poor prognosis in patients with ischemic stroke [45–46]. In our study, MPV/PLT and RDW/PLT were positively correlated with the risk of ischemic stroke in univariate comparisons. However, no statistically significant predictive value was observed in the combined analysis with different genotypes of MTHFR C677T and A1298C, which requires further research and verification.

In our ROC curve prediction model, the combination of MTHFR C677T, NE and MPV/LY showed modest predictive value for assessing the risk of ischemic stroke (AUC = 0.784, 95% CI: 0.726–0.842). This suggests that these novel integrated inflammation‑thrombosis biomarkers may help in rapidly identifying patients who could have more severe conditions or poorer prognosis at admission, thereby enhancing monitoring and treatment. However, we acknowledge that this AUC value is modest and that the model has not yet been validated in an independent external cohort; therefore, its clinical utility remains to be established.

Several limitations should be acknowledged. First, the sample size is relatively small (121 per group), and some combined genotype categories had zero counts in the control group, which may lead to unstable OR estimates. Second, the IS group was significantly older than the control group (mean difference 10 years). Although we adjusted for age in multivariate logistic regression, residual confounding cannot be fully excluded. Third, data on traditional vascular risk factors (hypertension, diabetes, dyslipidemia, smoking) were not collected due to the retrospective study design. Fourth, serum homocysteine, folate, and vitamin B12 levels were not measured; therefore, we cannot confirm that the observed associations between MTHFR polymorphisms and ischemic stroke risk are mediated through the homocysteine pathway. Our mechanistic references are based on the established literature rather than direct measurements in this cohort. Fifth, TOAST subtype classification was not performed due to the retrospective design and incomplete clinical records. Given that MTHFR polymorphisms have been reported to show stronger associations with small vessel stroke, the lack of subtype data may have introduced heterogeneity and potentially attenuated the observed effect sizes. Sixth, multiple comparisons were performed; we applied Bonferroni correction where indicated, but the possibility of type I errors remains. Seventh, the ROC analysis was performed on the same dataset used for model development, which may lead to overfitting; external validation in an independent cohort is essential. Finally, the Gansu region is multi‑ethnic, but sample size limitations precluded stratified analysis by ethnicity. Future studies with larger sample sizes and comprehensive covariate collection are needed to validate these findings and to explore potential ethnic differences.

In conclusion, this study demonstrates that *MTHFR* C677T and A1298C polymorphisms are independently associated with ischemic stroke risk in the Gansu population. Elevated MPV/LY and decreased PLT/NE ratios are also independent risk factors. A model combining NE, MPV/LY and *MTHFR* C677T genotyping showed modest predictive value (AUC = 0.784). Due to the lack of external validation and direct homocysteine measurements, these findings are hypothesis-generating and require confirmation in larger prospective cohorts.

## Supplementary Information

Supplementary Material 1.

## Data Availability

The datasets generated during the current study have been deposited in the NCBI ClinVar database and are publicly available. The accession numbers are SCV007601567 (for MTHFR C677T) and SCV007601568 (for MTHFR A1298C), with the following direct links: https://www.ncbi.nlm.nih.gov/clinvar. No additional data are available from the corresponding author beyond what has been deposited in the public database.

## Abbreviations

MTHFR: Methylenetetrahydrofolate Reductase
IS: Ischemic Stroke
MPV: Mean Platelet Volume
PLT: Platelet Count
LY: Lymphocyte Count
RDW: Red Cell Distribution Width
NE: Neutrophil Count
OR: Odds Ratios
CI: Confidence Intervals
ROC: Receiver Operating Characteristic
AUC: Area Under the Curve
