# Supplementary material for: Association of MTHFR and platelet-related blood cell ratio parameters with ischemic stroke in Gansu population
Source: BMC Cardiovasc Disord. 2026 Jul 7;26:854. doi: 10.1186/s12872-026-06229-2 (PMC13625494; doi:10.1186/s12872-026-06229-2)
Supplement: Supplementary file 1 [file 12872_2026_6229_MOESM1_ESM.docx]

| TableS1. MTRR A66G Genotype Distribution | | | | |
| --- | --- | --- | --- | --- |
| Genotype |  | Case(%) | Control(%) | P-value |
| MTRR(66A>G) | AA | 68(56.20) | 51(42.15) | 0.086 |
|  | AG | 41(33.88) | 56(46.28) |  |
|  | GG | 12(9.92) | 14(11.57) |  |
|  | A | 177(73.14) | 158(65.29) | 0.061 |
|  | G | 65(26.86) | 84(34.71) |  |

| Table S2. Relationship between MTHFR C677T, A1298C and MTRR A66G Genotype Distribution and Gender | | | | | | | | | | | | | | |
| --- | --- | --- | --- | --- | --- | --- | --- | --- | --- | --- | --- | --- | --- | --- |
| Group | Gender | Number |  | MTHFR C677T | | |  | MTHFR A1298C | | |  | MTRR A66G | | |
|  |  |  |  | CC,% | CT,% | TT,% |  | AA,% | AC,% | CC,% |  | AA,% | AG,% | GG,% |
| Ischemic Stroke | Male | 79 |  | 15(18.99) | 27(34.18 ) | 37(46.83 ) |  | 50(63.29 ) | 20(25.32 ) | 9(11.39 ) |  | 49(62.03 ) | 23(29.11) | 7(8.86 ) |
|  | Female | 42 |  | 9(21.43 ) | 16(38.10 ) | 17(40.47 ) |  | 31(73.81 ) | 9(21.43) | 2(4.76 ) |  | 19(45.24 ) | 18(42.86 ) | 5(11.90 ) |
|  | Total | 121 |  | 24(19.83) | 43(35.54) | 54(44.63 ) |  | 81(66.94) | 29(23.97) | 11(9.09 ) |  | 68(56.20) | 41(33.88) | 12(9.92 ) |
| Control | Male | 65 |  | 21(32.31 ) | 28(43.07 ) | 16(24.62)* |  | 46(70.77 ) | 18(27.69 ) | 1(1.54 ) |  | 24(36.93 ) | 31(47.69 ) | 10(15.38 )* |
|  | Female | 56 |  | 8(14.28 ) | 31(55.36 ) | 17(30.36 ) |  | 41(73.21) | 14(25.00 ) | 1(1.79 ) |  | 27(48.22) | 25(44.64 ) | 4(7.14 ) |
|  | Total | 121 |  | 29(23.97) | 59(48.76) | 33(27.27 ) |  | 87(71.90) | 32(26.45) | 2(1.65 ) |  | 51(42.15) | 56(46.28) | 14(11.57 ) |

| Table S3. Relationship between MTRR Genotype Distribution and Relative Risk of Ischemic Stroke | | | | | |
| --- | --- | --- | --- | --- | --- |
| Variables | Biomarker |  | MTRR A66G | | |
|  |  |  | AA | AG | GG |
| Control | NE |  | 3.36±1.10 | 3.52±1.25 | 3.31±0.97 |
|  | LY |  | 1.85±0.73 | 1.61±0.45 | 1.80±0.57 |
|  | RDW |  | 43.76±2.85 | 44.18±2.69 | 43.43±2.47 |
|  | PLT |  | 206.49±44.74 | 202.12±58.33 | 199.29±46.70 |
|  | MPV |  | 10.45±1.34 | 10.51±1.29 | 10.61±1.93 |
|  | MPV/PLT |  | 0.05±0.02 | 0.06±0.02 | 0.06±0.03 |
|  | MPV/LY |  | 6.35±2.33 | 7.05±2.22 | 6.40±2.18 |
|  | PLT/NE |  | 67.57±25.42 | 63.11±28.22 | 64.88±23.08 |
|  | RDW/PLT |  | 0.22±0.06 | 0.24±0.10 | 0.23±0.08 |
| Ischemic stroke | NE |  | 5.75±3.99 | 5.38±2.78 | 5.44±3.82 |
|  | LY |  | 1.52±0.74 | 1.25±0.57 | 1.37±0.36 |
|  | RDW |  | 45.60±5.77 | 46.44±7.54 | 47.17±5.80 |
|  | PLT |  | 193.34±70.07 | 190.66±76.66 | 191.75±57.57 |
|  | MPV |  | 11.38±1.11 | 11.36±1.22 | 11.34±1.01 |
|  | MPV/PLT |  | 0.07±0.04 | 0.07±0.03 | 0.07±0.03 |
|  | MPV/LY |  | 9.86±6.52 | 11.19±5.90 | 8.87±2.82 |
|  | PLT/NE |  | 45.15±28.03 | 45.30±26.72 | 47.98±31.60 |
|  | RDW/PLT |  | 0.27±0.13 | 0.29±0.16 | 0.26±0.07 |

| Table S4. Different genotype combinations of MTHFR C677T 、MTHFR A1298C and MTRR A66G polymorphisms in Ischemic Stroke and control groups. | | | | |
| --- | --- | --- | --- | --- |
| Genotypes | Control （ n (%) ） | Ischemic Stroke（ ( n (%)） | OR (95% CI) | P |
| CC/AA/AA | 6 | 5 | 1.000 (reference) | - |
| CC/AA/AG | 7 | 5 | 0.857(0.164-4.467) | 0.855 |
| CC/AA/GG | 1 | 0 | 0.857(0.633-1.160) | 0.377 |
| CC/AC/AA | 7 | 6 | 1.029(0.205-5.154) | 0.973 |
| CC/AC/AG | 4 | 5 | 1.500(0.255-8.817) | 0.653 |
| CC/AC/GG | 2 | 0 | 0.750(0.503-1.119) | 0.224 |
| CC/CC/AA | 0 | 1 | 1.200(0.839-1.716) | 0.296 |
| CC/CC/AG | 2 | 2 | 1.200(0.121-11.865) | 0.876 |
| CC/CC/GG | 0 | 0 | - | - |
| CT/AA/AA | 17 | 15 | 1.059(0.268-4.188) | 0.935 |
| CT/AA/AG | 20 | 9 | 0.540(0.130-2.243) | 0.393 |
| CT/AA/GG | 3 | 4 | 1.600(0.237-10.809) | 0.629 |
| CT/AC/AA | 8 | 8 | 1.200(0.257-5.593) | 0.816 |
| CT/AC/AG | 8 | 3 | 0.450(0.076-2.669) | 0.375 |
| CT/AC/GG | 3 | 0 | 0.667(0.420-1.058) | 0.145 |
| CT/CC/AA | 0 | 2 | 1.200-(0.121-11.865) | 0.876 |
| CT/CC/AG | 0 | 2 | 1.200-(0.121-11.865) | 0.876 |
| CT/CC/GG | 0 | 0 | - | - |
| TT/AA/AA | 13 | 24 | 2.215(0.566-8.678) | 0.248 |
| TT/AA/AG | 15 | 11 | 0.880(0.213-3.637) | 0.86 |
| TT/AA/GG | 5 | 8 | 1.920(0.376-9.800) | 0.431 |
| TT/AC/AA | 0 | 5 | 2.000(1.076-3.717) | 0.037* |
| TT/AC/AG | 0 | 2 | 1.200-(0.121-11.865) | 0.876 |
| TT/AC/GG | 0 | 0 | - | - |
| TT/CC/AA | 0 | 2 | 1.200-(0.121-11.865) | 0.876 |
| TT/CC/AG | 0 | 2 | 1.200-(0.121-11.865) | 0.876 |
| TT/CC/GG | 0 | 0 | - | - |
| *P-value from Fisher's exact test (for zero cells) or chi-square test. Nominal significance; does not survive Bonferroni correction for 23 comparisons. | | | | |

| Table S5. Primer and probe sequences for genotyping | | | |  |
| --- | --- | --- | --- | --- |
| Locus | Primer/Probe | Sequence (5′→3′) | Concentration (nM） |  |
| MTHFR C677T | Forward primer | TGAGGAGCTGTGAGGAGTA | 200 |  |
|  | Reverse primer | AGTCCAGCAAGGGAGAA | 200 |  |
|  | Probe (C allele) | FAM-ATGAAATCGGCTCCCGCAGAC-BHQ1 | 100 |  |
|  | Probe (T allele) | HEX-ATGAAATCGACTCCCGCAGAC-BHQ1 | 100 |  |
| MTHFR A1298C | Forward primer | CTTTGGGGAGCTGAAGGACTA | 200 |  |
|  | Reverse primer | CACTTTGTGACCATTCCGGT | 200 |  |
|  | Probe (A allele) | FAM-AGCAAGTCCCCCAAGGAGG-BHQ1 | 100 |  |
|  | Probe (C allele) | HEX-AGCAAGTCCCCCAAGGAGC-BHQ1 | 100 |  |
| MTRR A66G | Forward primer | GGCATAGTTCCAAAAGCCATC | 200 |  |
|  | Reverse primer | GTACAGGCATCCATGGATCT | 200 |  |
|  | Probe (A allele) | FAM-TGCAGCCATGCTTCTCTACATGG-BHQ1 | 100 |  |
|  | Probe (G allele) | HEX-TGCAGCCGTGCTTCTCTACATGG-BHQ1 | 100 |  |
| Internal control (β-globin) | Forward primer | GAAGAGCCAAGGACAGGTAC | 100 |  |
|  | Reverse primer | CAACTTCATCCACGTTCACC | 100 |  |
|  | Probe | CY5-GGTTGGCCAATCTACTCCCAGG-BHQ2 | 100 |  |
| Internal control gene: β-globin Reproducibility: 10% of samples were randomly selected and re-genotyped, with 100% concordance. Thermal cycling conditions: 95°C for 3 min; 40 cycles of 95°C for 15 sec, 60°C for 30 sec. | | | |  |
|  |  |  |  |  |

| Table S6. Quality control data for genotyping | | | |
| --- | --- | --- | --- |
| Parameter | MTHFR C677T | MTHFR A1298C | MTRR A66G |
| Call rate (%) | 99.2 | 98.8 | 99.2 |
| Reproducibility (%) | 100 | 100 | 100 |
| HWE P-value (controls) | 0.23 | 0.41 | 0.38 |
| HWE P-value (cases) | 0.19 | 0.35 | 0.42 |
| HWE: Hardy-Weinberg equilibrium. | | | |

| Table S7. Hardy-Weinberg equilibrium test results of each gene locus in case group and control group | | | | | | | |
| --- | --- | --- | --- | --- | --- | --- | --- |
| Gene Locus | Group | Genotype Distribution | | | χ² | P-value | Consistent with HWE |
| MTHFR C677T | Control | 29(CC) | 59(CT) | 33(TT) | 0.068 | >0.05 | Yes |
| MTHFR C677T | IS | 24(CC) | 43(CT) | 54(TT) | 7.13 | >0.05 | Yes |
| MTHFR A1298C | Control | 81(AA) | 29(AC) | 11(CC) | 9.455 | >0.05 | Yes |
| MTHFR A1298C | IS | 87(AA) | 32(AC) | 2(CC) | 0.237 | >0.05 | Yes |
| MTRR A66G | Control | 68(AA) | 41(AG) | 12(GG) | 2.291 | >0.05 | Yes |
| MTRR A66G | IS | 51(AA) | 56(AG) | 14(GG) | 0.054 | >0.05 | Yes |
